# Neoadjuvant talazoparib in patients with germline *BRCA1/2* mutation-positive, early-stage triple-negative breast cancer: exploration of tumor *BRCA* mutational status

**DOI:** 10.1007/s12282-024-01603-4

**Published:** 2024-06-13

**Authors:** Melinda L. Telli, Jennifer K. Litton, J. Thaddeus Beck, Jason M. Jones, Jay Andersen, Lida A. Mina, Raymond Brig, Michael Danso, Yuan Yuan, William F. Symmans, Julia F. Hopkins, Lee A. Albacker, Antonello Abbattista, Kay Noonan, Marielena Mata, A. Douglas Laird, Joanne L. Blum

**Affiliations:** 1 Department of Medicine, Stanford University School of Medicine, Stanford, CA USA; 2 Department of Breast Medical Oncology, The University of Texas MD Anderson Cancer Center, Houston, TX USA; 3 Department of Medical Oncology and Hematology, Highlands Oncology, Springdale, AR USA; 4 Avera Medical Group Oncology & Hematology, Avera Cancer Institute, Sioux Falls, SD USA; 5 Medical Oncology, Compass Oncology, West Cancer Center, US Oncology Network, Tigard, OR USA; 6 Hematology Oncology Department, Banner MD Anderson Cancer Center, Gilbert, AZ USA; 7 Medical Oncology, Brig Center for Cancer Care and Survivorship, Knoxville, TN USA; 8 Medical Oncology, Virginia Oncology Associates, Norfolk, VA USA; 9 Department of Medical Oncology & Therapeutics Research, Cedars-Sinai Cancer Center, West Hollywood, CA USA; 10 Department of Pathology, The University of Texas MD Anderson Cancer Center, Houston, TX USA; 11 Foundation Medicine, Inc., Cambridge, MA USA; 12 Clinical Statistics, Pfizer Oncology, Milan, Italy; 13 Clinical Oncology, Pfizer Inc., Groton, CT USA; 14 Pfizer Inc., La Jolla, CA USA; 15 Pfizer Inc., South San Franciso, CA USA; 16 Department of Oncology, Texas Oncology-Baylor Charles A. Sammons Cancer Center, US Oncology Network, Dallas, TX USA

**Keywords:** Talazoparib, Neoadjuvant, *BRCA1*, *BRCA2*, Triple-negative breast cancer

## Abstract

**Background:**

Talazoparib monotherapy in patients with germline *BRCA*-mutated, early-stage triple-negative breast cancer (TNBC) showed activity in the neoadjuvant setting in the phase II NEOTALA study (NCT03499353). These biomarker analyses further assessed the mutational landscape of the patients enrolled in the NEOTALA study.

**Methods:**

Baseline tumor tissue from the NEOTALA study was tested retrospectively using FoundationOne^®^CDx. To further hypothesis-driven correlative analyses, agnostic heat-map visualizations of the FoundationOne^®^CDx tumor dataset were used to assess overall mutational landscape and identify additional candidate predictive biomarkers of response.

**Results:**

All patients enrolled (*N* = 61) had TNBC. In the biomarker analysis population, 75.0% (39/52) and 25.0% (13/52) of patients exhibited *BRCA1* and *BRCA2* mutations, respectively. Strong concordance (97.8%) was observed between tumor *BRCA* and germline *BRCA* mutations, and 90.5% (38/42) of patients with tumor *BRCA* mutations evaluable for somatic-germline-zygosity were predicted to exhibit *BRCA* loss of heterozygosity (LOH). No patients had non-*BRCA* germline DNA damage response (DDR) gene variants with known/likely pathogenicity, based on a panel of 14 non-*BRCA* DDR genes. Ninety-eight percent of patients had *TP53* mutations. Genomic LOH, assessed continuously or categorically, was not associated with response.

**Conclusion:**

The results from this exploratory biomarker analysis support the central role of *BRCA* and *TP53* mutations in tumor pathobiology. Furthermore, these data support assessing germline *BRCA* mutational status for molecular eligibility for talazoparib in patients with TNBC.

**Supplementary Information:**

The online version contains supplementary material available at 10.1007/s12282-024-01603-4.

## Introduction

Germline pathogenic variants in *BRCA1* and *BRCA2* account for approximately 5% of all breast cancers and up to 30% of hereditary breast cancer. Germline *BRCA1/2* mutation carriers have a high incidence of breast cancer in their lifetime [1]; the lifetime probability is approximately 57–65% for those with *BRCA1* mutations and 45–49% for those with *BRCA2* mutations [2]. *BRCA1/2* mutations can undergo loss of heterozygosity (LOH), wherein the non-mutated allele loses function in parallel with the original identified mutation [3]. Women with germline *BRCA* mutations are more prone to developing breast cancer at a younger age and with more aggressive disease than those with somatic mutations [2].

Approximately 70% of breast tumors containing germline *BRCA1* mutations are diagnosed as triple-negative breast cancer (TNBC), a highly aggressive subtype of breast cancer associated with poor outcomes due to limited treatment options [2, 4]. A standard treatment approach for patients with early-stage TNBC is neoadjuvant chemotherapy, including patients with *BRCA1/2* mutations [4, 5]. The FDA has approved pembrolizumab plus neoadjuvant chemotherapy as standard of care for patients with high-risk early-stage TNBC [6]. In addition, several further trials have demonstrated that platinum-containing regimens were effective for TNBC in the neoadjuvant setting; however, the efficacy of neoadjuvant platinum for patients with *BRCA* mutations remains unclear [5, 7–10].

*BRCA1/2* mediate the repair of DNA double-strand breaks (DSBs) by homologous recombination repair (HRR) [11]. Cancer cells with germline *BRCA1/2* mutations rely on poly(ADP-ribose) polymerase (PARP) enzymes 1 and 2 for DNA repair [12]. Inhibition of single-strand DNA repair mechanisms mediated by PARP1/2 results in increased single-strand DNA breaks, which eventually culminate in the accumulation of DNA DSBs that are normally repaired via HRR [11, 13]. Hence, PARP inhibition is synthetically lethal in *BRCA*-mutated cancer cells, which are deficient in HRR [11]. Moreover, PARP inhibitors trap PARP to sites of DNA damage and prevent replication fork progression, resulting in irreparable DNA DSBs [11, 14]. Synthetic lethality therefore provides a mechanistic rationale for the treatment of *BRCA*-mutated breast cancer, including TNBC, with PARP inhibitors [15, 16]. In addition, pre-clinical models suggest that *BRCA* LOH may increase sensitivity to PARP inhibition [17].

Talazoparib is an oral PARP inhibitor approved for use in the US, EU, and multiple other countries as a monotherapy for the treatment of patients with deleterious or suspected deleterious germline *BRCA*-mutated human epidermal growth factor receptor 2 (HER2)-negative locally advanced or metastatic breast cancer [18, 19]. In the phase II NEOTALA study, talazoparib monotherapy in patients with germline *BRCA*-mutated, HER2-negative early breast cancer (Stage I–III; disease confined to the breast) including TNBC was active in the neoadjuvant setting (pathologic complete response [pCR] rate by independent central review [ICR] was 45.8% [95% confidence interval (CI), 32.0–60.6%] in the population evaluable for primary analysis on pCR) and showed a safety profile consistent with previous findings [20, 21].

The aim of the biomarker analyses reported here was to assess the mutational landscape of patients in the NEOTALA study and to explore potential associations of these factors with patient outcomes [22]. This was achieved by evaluating tumor tissue as well as blood and saliva samples from patients enrolled in the NEOTALA study with exploration of tumor *BRCA* mutations including germline-somatic concordance and zygosity; the non-*BRCA* germline DNA damage response (DDR) mutational landscape; homologous recombination deficiency (HRD) as assessed via genomic LOH; and the non-*BRCA* tumor mutational landscape. In addition, *TP53*, *MYC*, and *RAD21* genes were selected for exploratory analysis on pCR based on mutational prevalence, potential for crosstalk with DDR pathways [23, 24], and potential associations with outcome previously detected in the phase III EMBRACA study [25, 26].

## Patients and methods

### Study design and patients

NEOTALA (NCT03499353) was a phase II, non-randomized, single-arm, open-label study evaluating the efficacy and safety of talazoparib in the neoadjuvant setting for patients with early-stage germline *BRCA1/2-*mutated TNBC. Details of the primary study have been published [20, 21].

Briefly, eligible patients were ≥18 years old with histologically confirmed early HER2-negative carcinoma of the breast, germline *BRCA1/2* mutations, and no evidence of distant metastases. The primary endpoint was pCR by ICR after 24 weeks of neoadjuvant talazoparib treatment followed by surgery. Residual cancer burden (RCB) by ICR was a key secondary endpoint. RCB 0 refers to no residual invasive cancer, or pCR [27]. RCB I refers to minimal RCB, RCB II to moderate RCB, and RCB III to extensive RCB (progressive disease) [28].

### Mutational analysis

To assess molecular eligibility for the NEOTALA study, peripheral blood was tested using BRACAnalysis CDx^®^ (Myriad Genetics Laboratories, Inc., Salt Lake City, UT, USA) [20, 21]. In the analyses reported here, baseline tumor tissue was tested retrospectively using FoundationOne^®^CDx (Foundation Medicine, Inc., Cambridge, MA, USA), which is a qualitative next-generation sequencing (NGS)-based in vitro diagnostic test that uses targeted high throughput hybridization-based capture technology for detection of substitutions, insertion, and deletion alterations (indels), and copy number alterations (CNAs) in 324 genes and select gene rearrangements. *BRCA1/2* zygosity was assessed using somatic-germline-zygosity (SGZ) [29], and germline versus tumor sequence comparisons were used to determine whether tumor *BRCA* mutations were of germline or somatic origin. Tumor genomic LOH (gLOH) was assessed as an exploratory metric of HRD as previously described [30–32].

Germline mutational status of 14 non-*BRCA* DDR genes (*ATM*, *BAP1*, *BARD1*, *BRIP1*, *CHEK2*, *FANCC*, *MLH1*, *MRE11A*, *NBN*, *PALB2*, *RAD50*, *RAD51C*, *RAD51D*, and *XRCC2*) was retrospectively assessed in baseline saliva samples using CustomNext^®^-Cancer (Ambry Genetics, CA, USA). For exploratory tumor and germline analyses, mutations were defined as known/likely pathogenic/deleterious single-nucleotide variants, insertions, deletions, rearrangements, or amplifications, unless otherwise stated. For analysis of prevalence of non-*BRCA* tumor mutations, CNAs were analyzed both collectively with other mutations and separately as these alterations often reflect larger genomic changes that are not necessarily associated with a specific gene [33, 34].

### Patient population and biomarker analysis

The biomarker analysis populations included all patients from the NEOTALA study intent-to-treat (ITT) population with tumor samples suitable for analysis by NGS. The total number of patients evaluable for exploratory biomarker analyses varied depending on the assay (i.e., mutation detection, zygosity prediction for detected mutations [limited to short variants in tumors of sufficient quality and purity], or gLOH) and matrix (tumor or saliva). Agnostic heat-map visualizations of the FoundationOne^®^CDx tumor dataset were used to assess the overall mutational landscape and to identity candidate predictive biomarkers.

### Efficacy endpoints

The association between the mutational status of *TP53*, *MYC*, *RAD21*, or *RB1* and the primary endpoint pCR as per ICR was investigated using logistic regression. In addition, the association between the mutational status of these genes and RCB was explored. RCB index was assessed as a continuous variable [28] in this case rather than categorically, to facilitate this correlative analysis.

## Results

### Patients

A total of 61 female patients with TNBC and an average age of 44.6 years were enrolled in the ITT analysis and safety population.

### Non-*BRCA* DDR germline landscape

Forty-nine patients were included in the population evaluable for non-*BRCA* germline DDR mutations. Variants of ambiguous or unknown significance were detected in the saliva of 3 (6.1%) patients, each for *PALB2* and *RAD50*; 2 (4.1%) patients for *ATM*; and 1 (2.0%) patient, each for *BAP1*, *BARD1*, *BRIP1*, *FANCC*, and *RAD51C* (Supplementary Table S1). No patients had known/likely pathogenic non-*BRCA* germline DDR variants.

### *BRCA* mutations and zygosity

**Fig. 1:**
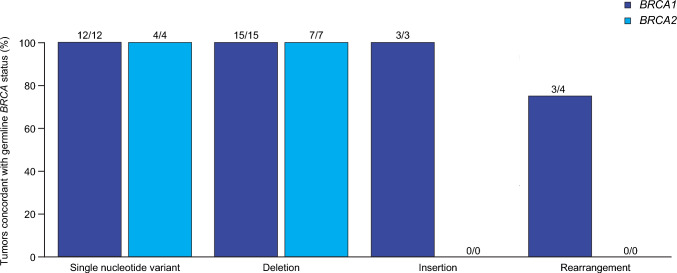
Percentage of tumors concordant with germline *BRCA1* or *BRCA2* mutational status. The proportion of patients with a known germline *BRCA1* mutation per Myriad Genetics laboratory results who had a *BRCA1* mutation (defined as known or likely pathogenic variant) detected in tumor using FoundationOne®CDx is shown, and likewise for *BRCA2*. One patient was discordant for *BRCA* mutational status (germline *BRCA1* exon 13 ins 6 kb [large rearrangement]) but exhibited no *BRCA1/2* mutation in tumor. All patients showing concordant *BRCA1* or *BRCA2* mutational status exhibited the same mutation in tumor as originally detected in germline

Of 52 tumor samples from the population evaluable by FoundationOne^®^CDx, 39 (75.0%) and 13 (25.0%) patients exhibited *BRCA1* and *BRCA2* mutations, respectively; 1 (1.9%) patient exhibited mutations in both genes; and 1 (1.9%) patient had mutations in neither (Supplementary Table S2). Among the 45 patients evaluable for both germline and tumor analyses, 44 (97.8%) exhibited the same *BRCA* mutation in tumor as originally detected in germline. The remaining patient exhibited a germline *BRCA1* rearrangement but lacked a tumor *BRCA* (t*BRCA*) mutation (Fig. 1). In the population evaluable for SGZ, 38/42 (90.5%) patients with t*BRCA* mutations were predicted to exhibit *BRCA* LOH (Table 1). Twenty-nine (69.0%) patients were predicted to have one or more *BRCA1* mutation with LOH and 9 (21.4%) were predicted to have one or more *BRCA2* mutation with LOH. Of the four patients not exhibiting *BRCA* LOH, two exhibited heterozygous t*BRCA2* mutations, one exhibited a heterozygous t*BRCA1* mutation, and one exhibited a heterozygous t*BRCA2* mutation and a t*BRCA1* rearrangement not amenable to SGZ prediction.

**Table 1:** Patients with tumor *BRCA* mutations predicted to exhibit LOH

Number of patients evaluable for *BRCA* zygosity, *n* (%)	42
At least one *BRCA1* mutation with LOH	29 (69.0)
At least one *BRCA2* mutation with LOH	9 (21.4)
No *BRCA1* or *BRCA2* mutations with LOH	4 (9.5)

LOH is predicted by somatic-germline-zygosity analysis (Foundation Medicine). LOH can refer to either copy-neutral LOH status (i.e., homozygous, both alleles carry the same variant in the tumor) or hemizygous status (i.e., loss of one allele in the tumor). There were 51 patients who exhibited mutations in *BRCA1* and/or *BRCA2*

*LOH* loss of heterozygosity

### Non-*BRCA* tumor mutational landscape

**Fig. 2:**
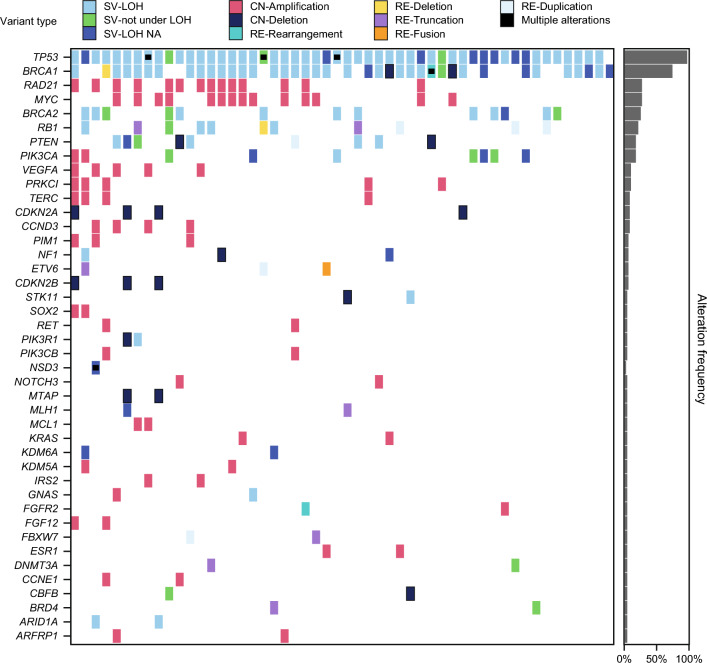
Tumor mutational landscape – population evaluable by FoundationOne®CDx. The figure displays only those genes altered in two or more patients. For rearrangements, if a partner gene was present, both genes were labeled. If a patient has multiple alterations in a gene, this is indicated by the addition of a black square on the tile. The tile background color depends on the SV-LOH status determined in the following order: SV-LOH, SV-not under LOH, SV NA, or if neither of the alterations are SVs, the tile will be colored as a CN or RE. For example, (i) a patient with one SVLOH and one SV-not under LOH will be colored light blue, (ii) two SVs not under LOH will be colored light green, (iii) a patient with one SV and one CN will be colored as determined by its SV LOH status. *CN* copy number, *LOH* loss of heterozygosity, *NA* not available, *RE* rearrangement, *SV* short variant

The overall tumor mutational landscape in the evaluable population is shown in Fig. 2. Based on variants with known or likely pathogenic impact, *TP53* mutations were near universal across all patients with one exception (*n* = 51; 98.1%). Genes with mutations having ≥10% prevalence included *BRCA1* (*n* = 39; 75.0%), *MYC* (*n* = 14; 26.9%), *RAD21* (*n* = 14; 26.9%), *BRCA2* (*n* = 13; 25.0%), *RB1* (*n* = 11; 21.2%), *PTEN* (*n* = 10; 19.2%), and *PIK3CA* (*n* = 9; 17.3%) (Table 2). Excluding CNAs, non-*BRCA* genes with a mutational prevalence ≥10% included *RB1* (*n* = 11; 21.2%), *PIK3CA* (*n* = 7; 13.5%), and *PTEN* (*n* = 7; 13.5%) (Table 2). In this TNBC subpopulation, *MYC* (14 [26.9%] patients) and *RAD21* (14 [26.9%] patients) were the most frequently mutated genes based on copy number changes with known or likely pathogenic impact (Table 2). In this study, despite the higher prevalence of *BRCA1* versus *BRCA2* mutations, *PIK3CA* tended to be more frequently co-mutated with *BRCA2* (*n* = 5/13) than with *BRCA1* (*n* = 4/39).

**Table 2:** Most prevalent (≥5.0%) tumor mutations

	Excluding CNAs *n* (%)	CNAs only *n* (%)	Any mutation
Patients in the population evaluable by FoundationOne^®^CDx, *n* (%)	52	52	52
*TP53*	51 (98.1)	0	51 (98.1)
*BRCA1*	37 (71.2)	3 (5.8)	39 (75.0)
*MYC*	0	14 (26.9)	14 (26.9)
*RAD21*	0	14 (26.9)	14 (26.9)
*BRCA2*	13 (25.0)	0	13 (25.0)
*RB1*	11 (21.2)	0	11 (21.2)
*PTEN*	7 (13.5)	5 (9.6)	10 (19.2)
*PIK3CA*	7 (13.5)	2 (3.8)	9 (17.3)
*PRKCI*	0	5 (9.6)	5 (9.6)
*VEGFA*	0	5 (9.6)	5 (9.6)
*CCND3*	0	4 (7.7)	4 (7.7)
*CDKN2A*	0	4 (7.7)	4 (7.7)
*TERC*	0	4 (7.7)	4 (7.7)
*BRD4*	2 (3.8)	1 (1.9)	3 (5.8)
*CDKN2B*	0	3 (5.8)	3 (5.8)
*ETV6*	3 (5.8)	0	3 (5.8)
*NF1*	2 (3.8)	1 (1.9)	3 (5.8)
*PIM1*	0	3 (5.8)	3 (5.8)

*CNA* copy number alteration

An exploratory analysis of the association between the tumor mutational status of *TP53*, *MYC*, and *RAD21* and pCR (ICR: breast/axilla dataset) is presented in Supplementary Table S3. The mutational status of *MYC* or *RAD21* had no observed association with pCR (for each *MYC* and *RAD21*: odds ratio = 0.52; 95% CI, 0.12–2.30; *p* = 0.3893). The mutational status of *TP53* had no observed association with pCR (*p* = 0.9629), although this analysis was confounded by the numerical imbalance favoring *TP53* mutations (Supplementary Table S3). Likewise, there was no apparent association between the mutational status of these genes and RCB scores (Supplementary Fig. S1).

**Fig. 3:**
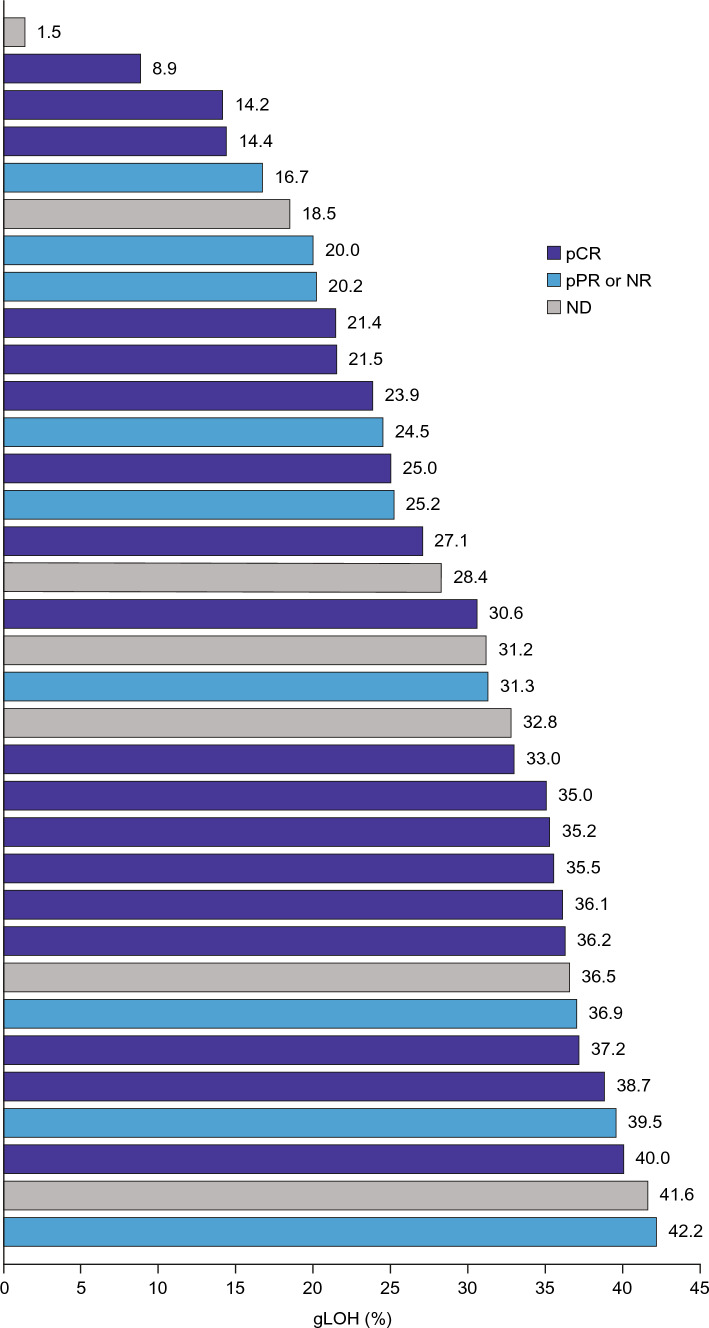
gLOH distribution – population evaluable for gLOH. *gLOH* genomic loss of heterozygosity, *ND* not determined, *NR* no response, *pCR* pathologic complete response, *pPR* pathologic partial response

A joint visualization of tumor gLOH status and pCR (ICR: breast/axilla dataset) is provided in Fig. 3 showing gLOH distribution ranges from 1.5 to 42.2% for evaluable patients, with no apparent relationship of gLOH score to response, and no signs of any potential threshold gLOH score distinguishing responders from non-responders. An analysis of response employing a gLOH threshold of ≥16% (which is predictive of biallelic *BRCA* loss in ovarian and breast cancer [30]) found that gLOH was elevated in 24/27 (88.9%) tumors evaluable for both gLOH and pCR. Consistent with the results displayed in Fig. 3, no association between tumor gLOH status and pCR was observed but this was confounded by the imbalance in prevalence favoring gLOH-high (odds ratio of gLOH-high vs. gLOH-low = not evaluable; *p* = 0.9447) (Supplementary Table S4).

### Exploration of potential associations of tumor mutations in other genes with response

**Fig. 4:**
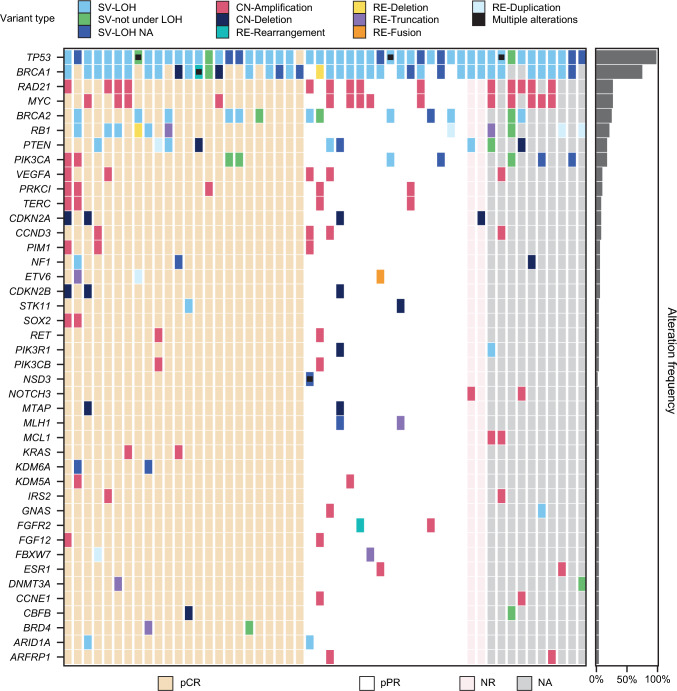
Tumor mutations by response category – evaluable biomarker analysis population. *CN* copy number, *LOH* loss of heterozygosity, *NA* not available, *NR* no response, *pCR* pathologic complete response, *pPR* pathologic partial response, *RE* rearrangement, *SV* short variant

To explore additional potential associations of tumor mutational status with outcome, an agnostic heat-map visualization of response as a function of mutational status was performed in the evaluable biomarker population (Fig. 4). In general, despite a low number of patients, mutations in most genes appeared to exhibit a similar distribution across response categories. For example, no association was apparent between *PIK3CA* and/or *PTEN* mutational status and response category. One potential exception was *RB1*, in which 6/11 (54.5%) patients with alterations had pCRs by ICR versus 1/11 (9.1%) patients with a pathologic partial response by ICR (Fig. 4), although the mutational status of *RB1* was not found to be significantly associated with pCR (odds ratio = 5.67; 95% CI, 0.62–52.09; *p* = 0.1254) (Supplementary Table S3). Similarly, RCB scores were typically lower for *RB1*-mutant patients in the population with tumors evaluable for FoundationOne^®^CDx and RCB analysis (Supplementary Fig. S1).

## Discussion

In this biomarker analysis of the NEOTALA study, tumor *BRCA* mutations were evident in all but one patient. Strong concordance (97.8%) was observed between tumor *BRCA* and germline *BRCA* mutations analyzed by FoundationOne^®^CDx and BRACAnalysis CDx^®^, respectively, except for one patient who exhibited a germline *BRCA1* mutation but lacked a tumor *BRCA* mutation. This exception could reflect genomic instability in that the mutation may have been lost [35], or alternatively, this germline rearrangement not being detectable in the tumor by FoundationOne^®^CDx. The strong concordance observed in this study is consistent with the findings in the phase III EMBRACA, phase II ABRAZO, and phase III OlympiAD studies in which 95.3%, 96.4%, and 99.3% of patients, respectively, exhibited concordance between tumor and germline *BRCA* mutational status [26, 36, 37].

In this TNBC population, a total of 38/42 (90.5%) patients with tumor *BRCA* mutations evaluable for SGZ were predicted to exhibit *BRCA* LOH. Previous studies support a high prevalence of *BRCA1/2* mutational LOH in breast cancer [30, 38]. This high incidence of *BRCA* LOH coupled with a low overall population size precludes meaningful analysis of tumor *BRCA* zygosity on response. This finding contrasts with another study analyzing germline *BRCA1/2*-mutated breast cancer tumors [17]; a greater proportion of patients in this study had one or more *BRCA1* mutation with LOH (69.0%) than patients with one or more *BRCA2* mutation with LOH (21.4%). In this previous analysis, LOH was seen in 90.2% of *BRCA1* carriers and 54.3% of *BRCA2* carriers [17].

No patients had non-*BRCA* germline DDR gene variants with known/likely pathogenicity, which may reflect the small patient numbers in this study and indicate that patients were enrolled based on the presence of germline *BRCA* mutations. *PALB2* and *RAD50* were the most commonly occurring variants of ambiguous or unknown significance (each 6.1%). In this TNBC population, tumor *PIK3CA* mutations, excluding CNAs, had a prevalence of 13.5%, which aligns with previous findings that show *PIK3CA* is frequently mutated in breast cancer [39, 40] and is potentially an actionable target in the advanced setting [41].

In this study, tumor *TP53* mutations, based on variants with known or likely pathogenic impact, excluding CNAs, were near-universal (*n* = 51; 98.1%). This finding is consistent with the anticipated high prevalence of *TP53* mutations in TNBC [15, 42]. In the NEOTALA study, *MYC* and *RAD21* both exhibited CNAs in 26.9% of tumors, with no association with pCR or RCB. This joint high prevalence is consistent with *MYC* and *RAD21* being closely linked on amplicon 8q24 and frequently coamplified in *BRCA1*-mutated breast cancer [43]. In the best versus worst responder visualization followed by Cox regression analysis of the EMBRACA study, an association between *MYC* amplification and short overall survival (OS) was observed in patients with TNBC receiving talazoparib (hazard ratio [HR] = 1.88; 95% CI, 1.10–3.20); this association was not evident in patients with TNBC receiving chemotherapy (HR = 0.71; 95% CI, 0.30–1.64) [25]. This finding was of interest considering that *MYC* has previously been linked to DDR gene regulation [24, 44]. Carey et al. demonstrate that *MYC* expression directly activates homologous recombination in response to DNA damage and could be used to predict response of patients with TNBC to therapy with PARP inhibitor combinations [24]. No such association with OS was seen in EMBRACA for *RAD21* [25], a structural component of the cohesin complex, despite being closely linked chromosomally with *MYC* [43].

In the NEOTALA study, an agnostic heat-map visualization of response as a function of mutational status revealed that mutations in most genes appeared to exhibit a similar distribution across response categories. A weak association of *RB1* alterations with response was observed, although this did not reach statistical significance (*p* = 0.1254). This finding is interesting given that a previous study demonstrated that *RB1* can localize to DNA DSBs, thus promoting DNA end resection and homologous recombination [45]. Notably, all four short variants of *RB1* under LOH exhibited pCR, which is intriguing given a report demonstrating that *RB1* loss is associated with tumors that are extremely sensitive to platinum retreatment in ovarian cancer [46]. Further exploration of the findings from this ad hoc exploratory analysis is warranted.

Genomic LOH (an exploratory metric of HRD), assessed continuously or categorically, was not associated with response. However, it should be noted that this lack of association is in a germline *BRCA*-mutated context, with all but three evaluable patients gLOH-high (i.e., gLOH ≥ 16%), and therefore does not address the free-standing predictive utility of gLOH in TNBC. For example, a composite HRD score comprising gLOH, telomeric allelic imbalance, and large-scale state transitions identified TNBC tumors, including *BRCA1/2*-non-mutated, as being more likely to respond to platinum-containing therapy [47].

The limitations of the NEOTALA study have previously been reported [21]; however, the limitations of the current study include the following: some of the retrospective exploratory analyses involved small sample sizes and certain analyses were confounded due to imbalances in the presence or absence of particular biomarkers.

Overall, the results from this exploratory biomarker analysis of the NEOTALA study support the central role of *BRCA* mutations in tumor pathobiology in patients with germline *BRCA1/2*-mutated TNBC and the value of assessing germline *BRCA* mutational status for molecular eligibility for talazoparib in this indication.

### Supplementary Information

Below is the link to the electronic supplementary material.

Supplementary file1 (DOCX 56 KB)

## Data Availability

Upon request and subject to review, Pfizer will provide the data that support the findings of this study. Subject to certain criteria, conditions and exceptions, Pfizer may also provide access to the related individual de-identified participant data. See https://www.pfizer.com/science/clinical-trials/trial-data-and-results for more information.

## Abbreviations

CI: Confidence interval
CN: Copy number
CNA: Copy number alteration
DDR: DNA damage response
DSB: Double-strand break
EU: European Union
FDA: US Food and Drug Administration
gLOH: Genomic LOH
HR: Hazard ratio
HER2: Human epidermal growth factor receptor 2
HRD: Homologous recombination deficiency
HRR: Homologous recombination repair
ICR: Independent central review
ITT: Intent-to-treat
LOH: Loss of heterozygosity
NA: Not available
ND: Not determined
NR: No response
NGS: Next-generation sequencing
OS: Overall survival
PARP: Poly(ADP-ribose) polymerase
pCR: Pathologic complete response
pPR: Pathologic partial response
RCB: Residual cancer burden
RE: Rearrangement
SGZ: Somatic-germline-zygosity
SV: Short variant
TNBC: Triple-negative breast cancer
US: United States
