# Supplementary material for: Neoadjuvant talazoparib in patients with germline BRCA1/2 mutation-positive, early-stage triple-negative breast cancer: exploration of tumor BRCA mutational status
Source: Breast Cancer. 2024 Jun 13;31(5):886–97. doi: 10.1007/s12282-024-01603-4 (PMC11341741; doi:10.1007/s12282-024-01603-4)
Supplement: Supplementary file 1 — Supplementary file1 (DOCX 56 KB) [file 12282_2024_1603_MOESM1_ESM.docx]

**Neoadjuvant talazoparib in patients with germline *BRCA1/2* mutation-positive, early-stage triple-negative breast cancer: exploration of tumor *BRCA* mutational status**

*Breast Cancer Research and Treatment*

Melinda L. Telli^1^, Jennifer K. Litton^2^, J. Thaddeus Beck^3^, Jason M. Jones^4^, Jay Andersen^5^, Lida A. Mina^6^, Raymond Brig^7^, Michael Danso^8^, Yuan Yuan^9^, William F. Symmans^10^, Julia F. Hopkins^11^, Lee A. Albacker^11^, Antonello Abbattista^12^, Kay Noonan^13^, Marielena Mata^14^, A. Douglas Laird^14^, and Joanne L. Blum^15^

^1^Department of Medicine, Stanford University School of Medicine, Stanford, CA, USA

^2^Department of Breast Medical Oncology, The University of Texas MD Anderson Cancer Center, Houston, TX, USA

^3^Department of Medical Oncology and Hematology, Highlands Oncology, Springdale, AR, USA

^4^Avera Medical Group Oncology & Hematology, Avera Cancer Institute, Sioux Falls, SD, USA

^5^Medical Oncology, Compass Oncology, West Cancer Center, US Oncology Network, Tigard, OR, USA

^6^Hematology Oncology Department, Banner MD Anderson Cancer Center, Gilbert, AZ, USA

^7^Medical Oncology, Brig Center for Cancer Care and Survivorship, Knoxville, TN, USA

^8^Medical Oncology, Virginia Oncology Associates, Norfolk, VA, USA

^9^Department of Medical Oncology & Therapeutics Research, Cedars-Sinai Cancer Center, West Hollywood, CA, USA

^10^Department of Pathology, The University of Texas MD Anderson Cancer Center, Houston, TX, USA

^11^Foundation Medicine, Inc., Cambridge, MA, USA

^12^Clinical Statistics, Pfizer Oncology, Milan, Italy

^13^Clinical Oncology, Pfizer Inc., Groton, CT, USA

^14^Pfizer Inc., La Jolla, CA, USA

^15^Department of Oncology, Texas Oncology-Baylor Charles A. Sammons Cancer Center, US Oncology Network, Dallas, TX, USA

Corresponding author: Melinda L. Telli, Stanford University School of Medicine, Stanford Cancer Center, Stanford, CA 94305, USA. E-mail: mtelli@stanford.edu; phone: 650-724-9533; ORCID 0000-0001-7993-1235

**Supplementary Materials**

**Supplementary Table S1** Non-*BRCA* germline DDR mutation variants with unknown or ambiguous pathogenic impact

| **Patients in the evaluable biomarker population, *n* (%)** | *n* = 49 |
| --- | --- |
| *PALB2* | 3 (6.1) |
| *RAD50* | 3 (6.1) |
| *ATM* | 2 (4.1) |
| *BAP1* | 1 (2.0) |
| *BARD1* | 1 (2.0) |
| *BRIP1* | 1 (2.0) |
| *FANCC* | 1 (2.0) |
| *RAD51C* | 1 (2.0) |

Non-*BRCA* DDR genes analyzed included *ATM, BAP1, BARD1, BRIP1, CHEK2, FANCC, MLH1, MRE11A, NBN, PALB2, RAD50, RAD51C, RAD51D*, and *XRCC2*. No known/likely pathogenic variants were detected for these 14 non-*BRCA* genes.

*DDR* DNA damage response

**Supplementary Table S2** Tumor *BRCA* mutations

| **Patients evaluable for *BRCA* mutations, *n*** | 52 |
| --- | --- |
| **t*BRCA1* mutations, *n* (%)** | 39 (75.0) |
| Median number of *BRCA1* mutations per patient (min, max) | 1 (1, 2) |
| **t*BRCA2* mutations, *n* (%)** | 13 (25.0) |
| Median number of *BRCA2* mutations per patient (min, max) | 1 (1, 1) |
| **Both t*BRCA1/2* mutations, *n* (%)** | 1 (1.9) |
| Median number of *BRCA1/2* mutations per patient (min, max) | 2 (2, 2) |
| **No *BRCA* mutations, *n* (%)** | 1 (1.9) |

Percentages are calculated using the number of patients evaluable by FoundationOne®CDx as the denominator

**Supplementary Table S3** Associations between the tumor mutational status of *TP53*, *MYC*, *RAD21*, and *RB1* and pCR

|  | **pCR (*N* = 24)** | **pPR + NR (*N* = 18)** | **Association** |
| --- | --- | --- | --- |
| *TP53* |  |  |  |
| Mutant, *n* (%) | 23 (95.8) | 18 (100.0) |  |
| Wild-type, *n* (%) | 1 (4.2) | 0 |  |
| Odds ratio (95% CI)^a^ |  |  | NE |
| *p* value |  |  | 0.9629 |
| *MYC* |  |  |  |
| Mutant, *n* (%) | 4 (16.7) | 5 (27.8) |  |
| Wild-type, *n* (%) | 20 (83.3) | 13 (72.2) |  |
| Odds ratio (95% CI)^a^ |  |  | 0.52 (0.12–2.30) |
| *p* value |  |  | 0.3893 |
| *RAD21* |  |  |  |
| Mutant, *n* (%) | 4 (16.7) | 5 (27.8) |  |
| Wild-type, *n* (%) | 20 (83.3) | 13 (72.2) |  |
| Odds ratio (95% CI)^a^ |  |  | 0.52 (0.12–2.30) |
| *p* value |  |  | 0.3893 |
| *RB1, n* | 23 | 18 |  |
| Mutant, *n* (%) | 6 (26.1) | 1 (5.6) |  |
| Wild-type, *n* (%) | 17 (73.9) | 17 (94.4) |  |
| Odds ratio (95% CI)^a^ |  |  | 5.67 (0.62–52.09) |
| *p* value |  |  | 0.1254 |

Mutant status denotes detection of at least one known/likely pathogenic variant for the specified gene Wild-type status denotes variant not detected

*CI* confidence interval, *NE* not evaluable, *NR* no response, *pCR* pathological complete response, *pPR* pathological partial response.

^a^Odds ratio of mutant versus wild-type (reference) for *TP53, MYC, RAD21,* and *RB1*; 95% CI; and *p* values are calculated from logistic regression.

**Supplementary Table S4** Association between tumor gLOH status and pCR

|  | **pCR**  **(*N* = 24)** | **pPR + NR**  **(*N* = 18)** | **Association** |
| --- | --- | --- | --- |
| gLOH low/high, *n* | 18 | 9 |  |
| gLOH low | 3 (12.5) | 0 |  |
| gLOH high | 15 (62.5) | 9 (50.0) |  |
| Not determined | 6 (25.0) | 9 (50.0) |  |
| Odds ratio (95% CI)^a^ |  |  | NE |
| *p* value |  |  | 0.9447 |

Two patients had pathologic NR. Tumor gLOH was assessed using FoundationOne®CDx

*CI* confidence interval, *gLOH* genomic loss of heterozygosity, *NE* not evaluable, *NR* no response, *pCR* pathological complete response, *pPR* pathological partial response.

^a^Odds ratio of gLOH high versus gLOH low (reference) status, 95% CI, and *p* values are calculated from logistic regression. gLOH high/low is based on a cut-off ≥16%.

**Supplementary Fig S1** Residual cancer burden index by tumor mutational status of selected genes


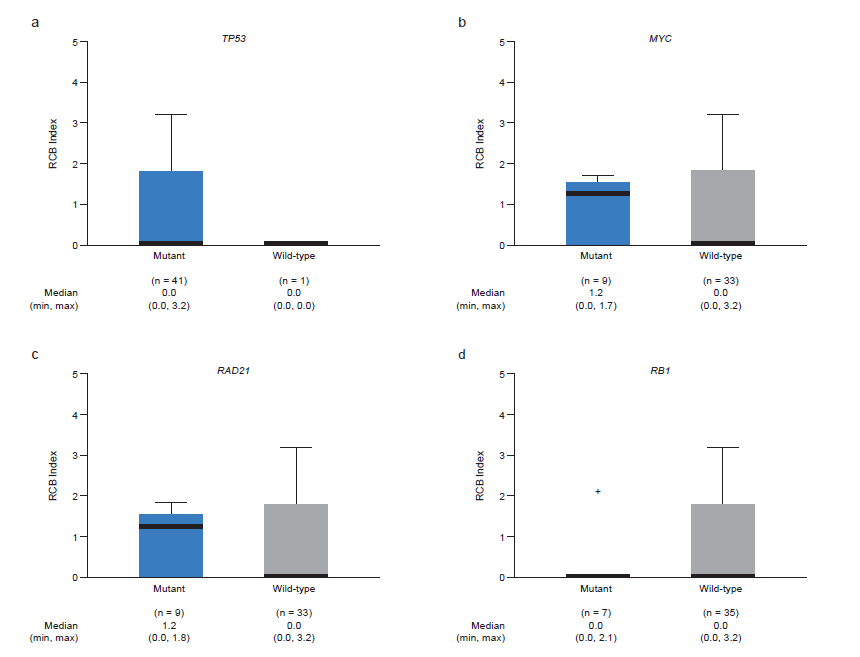


Results are based on tumor-evaluable population evaluable for RCB. Mutational status for six patients was not determined.

*max* maximum, *min* minimum, *RCB* residual cancer burden
